# Novel method for sub‐grouping of genotype II African swine fever viruses based on the intergenic region between the A179L and A137R genes

**DOI:** 10.1002/vms3.702

**Published:** 2021-12-29

**Authors:** Ha Thi Thanh Tran, Anh Duc Truong, Anh Kieu Dang, Duc Viet Ly, Nhu Thi Chu, Tuan Van Hoang, Huyen Thi Nguyen, Christopher L Netherton, Hoang Vu Dang

**Affiliations:** ^1^ Department of Biochemistry and Immunology National Institute of Veterinary Research Dong Da Hanoi Vietnam; ^2^ African Swine Fever Vaccinology Group The Pirbright Institute Surrey UK

**Keywords:** A137R, A179L, African swine fever, sub‐grouping, Vietnam

## Abstract

**Background:**

African swine fever (ASF) is a highly contagious and deadly viral disease affecting domestic and wild pigs of all ages. African swine fever virus (ASFV) has spread rapidly through Eastern and Southeastern Asia first appearing in Vietnam in 2019.

**Objectives:**

Molecular typing of African swine fever virus (ASFV) in Vietnam has identified two principal variants circulating based on the sequencing of the intergenic region (IRG) between the *I73R* and *I329L* genes. Identification of additional genetic markers would enable higher resolution tracing of outbreaks within the country.

**Methods:**

Sequence analysis suggested the IRG between the *A179L* and *A137R* genes may also exhibit variability, PCR primers were designed and samples from Vietnam were subject to Sanger sequencing.

**Results:**

We developed a novel method for sub‐grouping of ASFV based on the IRG between the *A179L* and *A137R* genes of ASFV. Our results demonstrated that the finding of the insertion or deletion of an 11‐ nucleotide sequence (GATACAATTGT) between the *A179L‐A137R* genes.

**Conclusions:**

The sub‐grouping method may provide useful insights into the evolution of genotype II ASFV as well as providing evidence of a relationship between geographically separated outbreaks.

## Graphical abstract

We report the development of a novel method for sub‐grouping genotype II African swine fever virus based on the intergenic region between the A179L and A137R genes. We have identified an insertion or deletion of an 11‐nucleotide sequence that may help analysis of the evolution and relationships between geographically separated outbreaks.

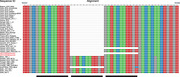

African swine fever (ASF) is a fatal viral disease that affects pigs of all ages and breeds that is reportable to the World Health Organization for Animal Health (OIE). ASF virus (ASFV) is highly virulent and remains a global threat because of the lack of a vaccine or drugs, and the ability of the virus to survive in various environmental conditions (Cubillos et al., 2013; Galindo & Alonso, 2017). Additionally, as a highly contagious virus, pigs infected with ASFV typically result in up to 100% of morbidity, and the mortality of ASF dependent on the virulence of the virus, the host, and transmission cycles (Kapoor et al., 2017; Quembo et al., 2018). Since the first confirmed outbreak of ASFV in China in 2018 the disease has spread to Mongolia, Vietnam, Cambodia, Hong Kong, Korea (Dem People's Rep. of), Laos, Myanmar, Philippines, Korea (Rep. of), Timor‐Leste, Indonesia, Papua New Guinea, India, Malaysia and Bhutan. ASF has also been reported on the Caribbean countries of Haiti and the Dominican Republic. From early February 2019, an ASF outbreak in Vietnam was reported officially, and within seven months, ASF had spread across the whole country. Approximately six million pigs on infected farms and households have been culled, indicating the socio‐economic impact on the pig industry (Tran et al., 2021; Truong et al., 2020).

Our previous reports that the ASFV strains circulating in Vietnam belong to *B646L*(p72) genotype II and *EP402R* (CD2v) serotype 8 and that there are least two different variants based on the intergenic region located between the *I73R* and *I329L* genes (Tran et al., 2021). Most investigations of ASFV genotypes have been identified based on sequencing of the *B646L* and *E183L* (p54) genes and/or analysis of the central variable region or intergenic region (IGR) between *I73R* and *I329L* genes (Gallardo et al., 2009; Gallardo et al., 2014). On the other hand, the recent research on the evolution of the *MGF505* family, *K145R*, and *O174L* genes of ASFV strains revealed minor genetic diversity within these genes, indicating slow but consistent molecular evolution of ASFV strains in a different region (Malogolovkin et al., 2015; Mazur‐Panasiuk et al., 2020). In this study, we developed a new method for ASFV sub‐grouping analysis of genotype II viruses based on the intergenic region between the *A179L* and *A137R* genes. Additionally, the data obtained from this marker region is useful to provide the evidence of a relationship between geographically separated outbreaks.

We have shown that two ASFV strains, ASFV/VN/Pig/Hanoi/02 and ASFV/VN/Pig/Hanoi/07, obtained from the Thach That district of Hanoi city, belonged to *B646L* genotype II, *EP402R*v serotype 8 and were closely related to strains circulating in China (Tran et al., 2021). Based on the IGR located between the *I73R* and *I329L* genes, four different variants of ASFVs have been reported, including IGR I, II, III and IV variants. Since the introduction of ASFV into Vietnam most viruses circulating in the country, including ASFV/VN/Pig/Hanoi/02 strain, belong to the p72 genotype II and IGR II variant. However, ASFV/VN/Pig/Hanoi/07 strain was classified as p72 genotype II ASFV and IGR I variant (Tran et al., 2021).

An alignment and analysis of the nucleotide sequences of ASFV strains from *B646L* genotype I and II revealed the presence of one to three copies of an 11‐nucleotide repeat (GATACAATTST) within the intergenic region between the *A179L* and *A137R* genes (Figure 1a, Supplementary Table S1). A pair of primers to amplify a 270‐bp fragment was designed based on the reference sequence and the primer binding sites were as follows: forward primer, 5′‐CCA TAG CGG CAC CCT ATA TT‐3′; reverse primer, 5′‐CCT CCT GGT CGA GTT TGG TA‐3′. The optimal annealing temperature was 50°C. PCR was performed, and the products were subjected to sequencing by the Sanger method. Lymph node samples from two dead pigs that the ASFV/VN/Pig/Hanoi/02 and ASFV/VN/Pig/Hanoi/07 viruses were originally isolated were used to investigate the *A179L‐A137R* intergenic region. Our results showed that an 11‐nucleotide deletion was found in ASFV/VN/Pig/Hanoi/07 isolate when compared to ASFV/VN/Pig/Hanoi/02 and other viruses isolated from Vietnam (Figure 1a). Importantly, a similar deletion was identified in an ASFV isolate from Vietnam that we had previously sequenced, ASFV_Hanoi_2019. In addition, the deletion was still present in Hanoi/07 after three passages through porcine alveolar macrophages. Further analysis revealed that the *A179L‐A137R* intergenic regions from forty‐five other genotype II ASFV sequences from Africa, Asia and Europe were identical to ASFV/VN/Pig/Hanoi/02, ASFV_NgheAn_2019 and VNUA‐ASFV‐05L1/HaNam/VN/2020 (Figure 1, Supplementary Table S1). Therefore, ASFV/VN/Pig/Hanoi07 and ASFV_Hanoi_2019 contain one copy of the GATACAATTST motif in the *A179L‐A137R* intergenic region, all the other available genotype II viruses have two copies of this motif and all p72 genotype I viruses, with the exception of one from South Africa, have three copies of the GATACATTST motif. This showed that the deletion within the *A179L‐A137R* intergenic regions found in ASFV/VN/Pig/Hanoi/07 and ASFV_Hanoi_2019 was novel. Future work will focus on whole genome sequencing of ASFV/VN/Pig/Hanoi/07 and ASFV/VN/Pig/Hanoi/02 as well as other ASFVs isolated in seven agricultural regions of Vietnam to get a better understanding of ASFV subgroups circulating in the country.

**FIGURE 1:**
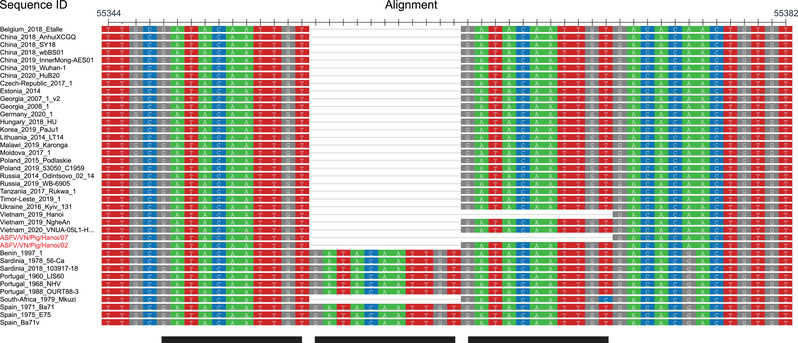
Alignment of the region between the *A179L* and *A137R* genes of the indicated p72 genotype I and II ASFV isolates. Alignment was prepared using MEGA7, and displayed using NCBI Multiple Sequence Alignment Viewer, Version 1.20.1. Numbers above the alignment show the positions relative to the Georgia 2007/1 isolate (FR682468.2), black bars indicate the GATACAATTGST repeats and the sequences from Hanoi/02 and Hanoi/07 are highlighted in red text

Based on these results, we conclude that the ASFV/VN/Pig/Hanoi/07 strain is a novel variant, belonging to the p72 genotype II, I73R/I329L variant I and A179L/A137R variant 1 (1 copy of the GATACAATTST motif), and differs from previously reported ASFV strains circulating in China and Vietnam. The genetic variability within the *A179L‐A137R* gene provides an opportunity for deeper insight into the spread of ASFV in Vietnam and provides evidence of a relationship between geographically separated outbreaks.

## AUTHOR CONTRIBUTIONS

Ha Tran: conceptualisation, formal analysis, investigation, methodology, software, writing – original draft. Duc Truong: conceptualisation, formal analysis, investigation, methodology, software, writing – original draft. Anh Dang: formal analysis, methodology, software. Viet Ly: formal analysis, methodology, software. Nhu Chu: formal analysis, methodology, software. Tuan Hoang: conceptualisation, investigation, methodology, writing – original draft. Huyen Nguyen: formal analysis, methodology, software. Christopher Netherton: formal analysis, investigation, methodology, visualisation, writing – original draft, writing – review & editing. Hoang Vu Dang: conceptualisation, funding acquisition, investigation, methodology, project administration, resources, supervision, writing – original draft, writing – review & editing.

## CONFLICT OF INTEREST

There are no potential conflicts of interest

## ETHICS STATEMENT

The study was conducted in compliance with the institutional rules for the care and use of laboratory animals and using a protocol approved by the Ministry of Agriculture and Rural Development (MARD) Vietnam (TCVN 8402:2010).

## Supporting information

Supporting information

## Data Availability

All sequences generated in this study were submitted to GenBank under accession nos. MW526931 (ASFV/VN/Pig/Hanoi/02) and MW526932 (ASFV/VN/Pig/Hanoi/07) for A179L‐A137R region genes.
