# Supplementary material for: Novel method for sub‐grouping of genotype II African swine fever viruses based on the intergenic region between the A179L and A137R genes
Source: Vet Med Sci. 2021 Dec 29;8(2):607–9. doi: 10.1002/vms3.702 (PMC8959334; doi:10.1002/vms3.702)
Supplement: Supplementary file 1 — Supporting information [file VMS3-8-607-s001.docx]

# Supplementary Materials

Supplementary Table 1: A179L-A137R intergenic sequences from genotype I and II viruses. This is provided in two formats, one ordered alphabetically by country and then by year within that country. The second is ordered by genotype and then by year.

## Sequences ordered by country and year of isolation

| **Accession #** | **Country** | **Year** | **Isolate Name** | **P72 Genotype** | **A179L-A137R variant** |
| --- | --- | --- | --- | --- | --- |
| LR536725 | Belgium | 2018 | Belgium/2018/1 | II | 2 |
| MK543947 | Belgium | 2018 | Belgium/Etalle/wb/2018 | II | 2 |
| AM712239 | Benin | 1997 | Benin 97/1 | I | 3 |
| MT496893 | China | 2018 | GZ201801 | II | 2 |
| MK128995 | China | 2018 | China/2018/AnhuiXCGQ | II | 2 |
| MK645909 | China | 2018 | ASFV-wbBS01 | II | 2 |
| MH766894 | China | 2018 | SY18 | II | 2 |
| MK333181 | China | 2018 | DB/LN/2018 | II | 2 |
| MH333180 | China | 2018 | Pig/HLJ/2018 | II | 2 |
| MK940252 | China | 2019 | CN/2019/InnerMongolia-AES01 | II | 2 |
| MN172368 | China | 2019 | CAS19-01/2019 | II | 2 |
| MN393476 | China | 2019 | Wuhan 2019-1 | II | 2 |
| MN393477 | China | 2019 | Wuhan 2019-2 | II | 2 |
| MW361944 | China | 2019 | China/GD/2019 | II | 2 |
| MW656282 | China | 2020 | Pig/Heilongjiang/HRB1/2020 | II | 2 |
| MW521382 | China | 2020 | HuB20 | II | 2 |
| LR722600 | Czech Republic | 2017 | CzechRepublic 2017/1 | II | 2 |
| LS478113 | Estonia | 2014 | Estonia 2014 | II | 2 |
| FR682468 | Georgia | 2007 | Georgia 2007/1 | II | 2 |
| MH910495 | Georgia | 2008 | Georgia 2008/1 | II | 2 |
| MH910496 | Georgia | 2008 | Georgia 2008/2 | II | 2 |
| LR899193 | Germany | 2020 | ASFV Germany 2020/1 | II | 2 |
| MN715134 | Hungary | 2018 | 2018_HU | II | 2 |
| MN270969 | Italy | 1978 | 56/Ca/1978 | I | 3 |
| MN270970 | Italy | 1979 | 57/Ca/1979 | I | 3 |
| MN270971 | Italy | 1981 | 139/Nu/1981 | I | 3 |
| MN270972 | Italy | 1985 | 140/Or/1985 | I | 3 |
| MN270973 | Italy | 1985 | 85/Ca/1985 | I | 3 |
| MN270974 | Italy | 1990 | 141/Nu/1990 | I | 3 |
| MN270975 | Italy | 1995 | 142/Nu/1995 | I | 3 |
| MN270976 | Italy | 1997 | 60/Nu/1997 | I | 3 |
| MN270977 | Italy | 2004 | 26/Ss/2004 | I | 3 |
| MN270978 | Italy | 2005 | 72407/Ss/2005 | I | 3 |
| KX354450 | Italy | 2008 | 47/Ss/2008 | I | 3 |
| KM102979 | Italy | 2010 | 26544/OG10 | I | 3 |
| MN270979 | Italy | 2012 | 97/Ot/2012 | I | 3 |
| MN270980 | Italy | 2014 | 22653/Ca/2014 | I | 3 |
| MT932579 | Italy | 2018 | 55234/18 | I | 3 |
| MT932578 | Italy | 2018 | 103917/18 | I | 3 |
| MT748042 | Korea | 2019 | Korea/pig/PaJu1/2019 | II | 2 |
| MK628478 | Lithuania | 2014 | LT14/1490 | II | 2 |
| MW856068 | Malawi | 2019 | MAL/19/Karonga | II | 2 |
| LR722599 | Moldova | 2017 | Moldova 2017/1 | II | 2 |
| MH681419 | Poland | 2015 | POL/2015/Podlaskie | II | 2 |
| MG939583 | Poland | 2016 | Pol16_20186_o7 | II | 2 |
| MG939584 | Poland | 2016 | Pol16_20538_o9 | II | 2 |
| MG939585 | Poland | 2016 | Pol16_20540_o10 | II | 2 |
| MG939586 | Poland | 2016 | Pol16_29413_o23 | II | 2 |
| MG939587 | Poland | 2017 | Pol17_03029_C201 | II | 2 |
| MG939588 | Poland | 2017 | Pol17_04461_C210 | II | 2 |
| MG939589 | Poland | 2017 | Pol17_05838_C220 | II | 2 |
| MT847620 | Poland | 2017 | Pol17_55892_C754 | II | 2 |
| MT847622 | Poland | 2017 | Pol17_31177_O81 | II | 2 |
| MT847621 | Poland | 2018 | Pol18_28298_O111 | II | 2 |
| MT847623 | Poland | 2019 | Pol19_53050_C1959/19 | II | 2 |
| KM262844 | Portugal | 1960 | L60 | I | 3 |
| KM262845 | Portugal | 1968 | NHV | I | 3 |
| AM712240 | Portugal | 1988 | OUR T88/3 | I | 3 |
| KP843857 | Russia | 2014 | Odintsovo_02/14 | II | 2 |
| MT459800 | Russia | 2019 | Karbardino-Balkaria/WB-964 | II | 2 |
| MW306190 | Russia | 2019 | Armur 19/WB-6905 | II | 2 |
| MW306191 | Russia | 2019 | Primorsky 19/WB-6723 | II | 2 |
| MW306192 | Russia | 2019 | AUlyanovsk 19/WB-5699 | II | 2 |
| AY261362 | South Africa | 1979 | Mkuzi 1979 | I | 2 |
| KP055815 | Spain | 1971 | Ba71 | I | 3 |
| FN557520 | Spain | 1975 | E75 | I | 3 |
| U18466 | Spain | NA | Ba71v | I | 3 |
| LR813622 | Tanzania | 2017 | Tanzania/Rukwa/2017/1 | II | 2 |
| MW39679 | Timor Leste | 2019 | Timor-Leste/2019/01 | II | 2 |
| MN194591 | Ukraine | 2016 | Kyiv/2016/131 | II | 2 |
| MT180393 | Vietnam | 2019 | NgheAn_2019 | II | 2 |
| MT166692 | Vietnam | 2019 | Hanoi_2019 | II | 1 |
| MW465755 | Vietnam | 2020 | VNUA-ASFV-05L1/HaNam/VN/2020 | II | 2 |
| MW526931 | Vietnam | 2020 | ASFV/VN/Pig/Hanoi/02 | II | 2 |
| MW526932 | Vietnam | 2020 | ASFV/VN/Pig/Hanoi/07 | II | 1 |

## Ordered by *B646L* genotype and year of isolation

| **Accession #** | **Country** | **Year** | **Isolate Name** | **P72 Genotype** | **A179L-A137R variant** |
| --- | --- | --- | --- | --- | --- |
| KM262844 | Portugal | 1960 | L60 | I | 3 |
| KM262845 | Portugal | 1968 | NHV | I | 3 |
| KP055815 | Spain | 1971 | Ba71 | I | 3 |
| FN557520 | Spain | 1975 | E75 | I | 3 |
| MN270969 | Italy | 1978 | 56/Ca/1978 | I | 3 |
| MN270970 | Italy | 1979 | 57/Ca/1979 | I | 3 |
| AY261362 | South Africa | 1979 | Mkuzi 1979 | I | 2 |
| MN270971 | Italy | 1981 | 139/Nu/1981 | I | 3 |
| MN270972 | Italy | 1985 | 140/Or/1985 | I | 3 |
| MN270973 | Italy | 1985 | 85/Ca/1985 | I | 3 |
| AM712240 | Portugal | 1988 | OUR T88/3 | I | 3 |
| MN270974 | Italy | 1990 | 141/Nu/1990 | I | 3 |
| MN270975 | Italy | 1995 | 142/Nu/1995 | I | 3 |
| AM712239 | Benin | 1997 | Benin 97/1 | I | 3 |
| MN270976 | Italy | 1997 | 60/Nu/1997 | I | 3 |
| MN270977 | Italy | 2004 | 26/Ss/2004 | I | 3 |
| MN270978 | Italy | 2005 | 72407/Ss/2005 | I | 3 |
| KX354450 | Italy | 2008 | 47/Ss/2008 | I | 3 |
| KM102979 | Italy | 2010 | 26544/OG10 | I | 3 |
| MN270979 | Italy | 2012 | 97/Ot/2012 | I | 3 |
| MN270980 | Italy | 2014 | 22653/Ca/2014 | I | 3 |
| MT932579 | Italy | 2018 | 55234/18 | I | 3 |
| MT932578 | Italy | 2018 | 103917/18 | I | 3 |
| U18466 | Spain | NA | Ba71v | I | 3 |
| FR682468 | Georgia | 2007 | Georgia 2007/1 | II | 2 |
| MH910495 | Georgia | 2008 | Georgia 2008/1 | II | 2 |
| MH910496 | Georgia | 2008 | Georgia 2008/2 | II | 2 |
| LS478113 | Estonia | 2014 | Estonia 2014 | II | 2 |
| MK628478 | Lithuania | 2014 | LT14/1490 | II | 2 |
| KP843857 | Russia | 2014 | Odintsovo_02/14 | II | 2 |
| MH681419 | Poland | 2015 | POL/2015/Podlaskie | II | 2 |
| MG939583 | Poland | 2016 | Pol16_20186_o7 | II | 2 |
| MG939584 | Poland | 2016 | Pol16_20538_o9 | II | 2 |
| MG939585 | Poland | 2016 | Pol16_20540_o10 | II | 2 |
| MG939586 | Poland | 2016 | Pol16_29413_o23 | II | 2 |
| MN194591 | Ukraine | 2016 | Kyiv/2016/131 | II | 2 |
| LR722600 | Czech Republic | 2017 | CzechRepublic 2017/1 | II | 2 |
| LR722599 | Moldova | 2017 | Moldova 2017/1 | II | 2 |
| MG939587 | Poland | 2017 | Pol17_03029_C201 | II | 2 |
| MG939588 | Poland | 2017 | Pol17_04461_C210 | II | 2 |
| MG939589 | Poland | 2017 | Pol17_05838_C220 | II | 2 |
| MT847620 | Poland | 2017 | Pol17_55892_C754 | II | 2 |
| MT847622 | Poland | 2017 | Pol17_31177_O81 | II | 2 |
| LR813622 | Tanzania | 2017 | Tanzania/Rukwa/2017/1 | II | 2 |
| LR536725 | Belgium | 2018 | Belgium/2018/1 | II | 2 |
| MK543947 | Belgium | 2018 | Belgium/Etalle/wb/2018 | II | 2 |
| MT496893 | China | 2018 | GZ201801 | II | 2 |
| MK128995 | China | 2018 | China/2018/AnhuiXCGQ | II | 2 |
| MK645909 | China | 2018 | ASFV-wbBS01 | II | 2 |
| MH766894 | China | 2018 | SY18 | II | 2 |
| MK333181 | China | 2018 | DB/LN/2018 | II | 2 |
| MH333180 | China | 2018 | Pig/HLJ/2018 | II | 2 |
| MN715134 | Hungary | 2018 | 2018_HU | II | 2 |
| MT847621 | Poland | 2018 | Pol18_28298_O111 | II | 2 |
| MK940252 | China | 2019 | CN/2019/InnerMongolia-AES01 | II | 2 |
| MN172368 | China | 2019 | CAS19-01/2019 | II | 2 |
| MN393476 | China | 2019 | Wuhan 2019-1 | II | 2 |
| MN393477 | China | 2019 | Wuhan 2019-2 | II | 2 |
| MW361944 | China | 2019 | China/GD/2019 | II | 2 |
| MT748042 | Korea | 2019 | Korea/pig/PaJu1/2019 | II | 2 |
| MW856068 | Malawi | 2019 | MAL/19/Karonga | II | 2 |
| MT847623 | Poland | 2019 | Pol19_53050_C1959/19 | II | 2 |
| MT459800 | Russia | 2019 | Karbardino-Balkaria/WB-964 | II | 2 |
| MW306190 | Russia | 2019 | Armur 19/WB-6905 | II | 2 |
| MW306191 | Russia | 2019 | Primorsky 19/WB-6723 | II | 2 |
| MW306192 | Russia | 2019 | AUlyanovsk 19/WB-5699 | II | 2 |
| MW39679 | Timor Leste | 2019 | Timor-Leste/2019/01 | II | 2 |
| MT180393 | Vietnam | 2019 | NgheAn_2019 | II | 2 |
| MT166692 | Vietnam | 2019 | Hanoi_2019 | II | 1 |
| MW656282 | China | 2020 | Pig/Heilongjiang/HRB1/2020 | II | 2 |
| MW521382 | China | 2020 | HuB20 | II | 2 |
| LR899193 | Germany | 2020 | ASFV Germany 2020/1 | II | 2 |
| MW465755 | Vietnam | 2020 | VNUA-ASFV-05L1/HaNam/VN/2020 | II | 2 |
| MW526931 | Vietnam | 2020 | ASFV/VN/Pig/Hanoi/02 | II | 2 |
| MW526932 | Vietnam | 2020 | ASFV/VN/Pig/Hanoi/07 | II | 1 |
